# Supplementary material for: Modeling glioblastoma heterogeneity as a dynamic network of cell states
Source: Mol Syst Biol. 2021 Sep 16;17(9):e10105. doi: 10.15252/msb.202010105 (PMC8444284; doi:10.15252/msb.202010105)
Supplement: Supplementary file 6 — Source Data for Figure 5 [file MSB-17-e10105-s004.zip › Figure5A_sourcedata/GSEA_3071/hallmarks_stateII.GseaPreranked.1623742536692/HALLMARK_E2F_TARGETS.html]

Details for gene set HALLMARK\_E2F\_TARGETS[GSEA]

|  || Dataset | state23071 |
| Phenotype | NoPhenotypeAvailable |
| Upregulated in class | na\_pos |
| GeneSet | HALLMARK\_E2F\_TARGETS |
| Enrichment Score (ES) | 0.71334183 |
| Normalized Enrichment Score (NES) | 4.5666385 |
| Nominal p-value | 0.0 |
| FDR q-value | 0.0 |
| FWER p-Value | 0.0 |
Table: GSEA Results Summary

  

Fig 1: Enrichment plot: HALLMARK\_E2F\_TARGETS      
 Profile of the Running ES Score & Positions of GeneSet Members on the Rank Ordered List

  

| PROBE | GENE SYMBOL | GENE\_TITLE | RANK IN GENE LIST | RANK METRIC SCORE | RUNNING ES | CORE ENRICHMENT || 1 | RRM2 |  |  | 3 | 1.857 | 0.0146 | Yes |
| 2 | CDK1 |  |  | 14 | 1.540 | 0.0249 | Yes |
| 3 | DIAPH3 |  |  | 15 | 1.531 | 0.0375 | Yes |
| 4 | BRCA2 |  |  | 22 | 1.445 | 0.0480 | Yes |
| 5 | RAD51AP1 |  |  | 25 | 1.384 | 0.0590 | Yes |
| 6 | MYBL2 |  |  | 28 | 1.362 | 0.0697 | Yes |
| 7 | USP1 |  |  | 31 | 1.357 | 0.0805 | Yes |
| 8 | TCF19 |  |  | 32 | 1.331 | 0.0915 | Yes |
| 9 | PSMC3IP |  |  | 36 | 1.322 | 0.1017 | Yes |
| 10 | ORC6 |  |  | 39 | 1.308 | 0.1120 | Yes |
| 11 | TK1 |  |  | 42 | 1.281 | 0.1222 | Yes |
| 12 | AURKB |  |  | 44 | 1.276 | 0.1325 | Yes |
| 13 | MCM7 |  |  | 45 | 1.273 | 0.1430 | Yes |
| 14 | HELLS |  |  | 46 | 1.259 | 0.1535 | Yes |
| 15 | BRCA1 |  |  | 49 | 1.249 | 0.1633 | Yes |
| 16 | GINS1 |  |  | 52 | 1.239 | 0.1731 | Yes |
| 17 | PCNA |  |  | 58 | 1.214 | 0.1819 | Yes |
| 18 | MAD2L1 |  |  | 61 | 1.199 | 0.1913 | Yes |
| 19 | UBE2T |  |  | 62 | 1.192 | 0.2012 | Yes |
| 20 | ATAD2 |  |  | 63 | 1.189 | 0.2111 | Yes |
| 21 | MSH2 |  |  | 66 | 1.163 | 0.2202 | Yes |
| 22 | RFC3 |  |  | 67 | 1.157 | 0.2298 | Yes |
| 23 | SPC25 |  |  | 68 | 1.156 | 0.2394 | Yes |
| 24 | MELK |  |  | 69 | 1.145 | 0.2489 | Yes |
| 25 | POLD3 |  |  | 70 | 1.143 | 0.2583 | Yes |
| 26 | TOP2A |  |  | 72 | 1.132 | 0.2675 | Yes |
| 27 | KIF2C |  |  | 73 | 1.132 | 0.2769 | Yes |
| 28 | MCM5 |  |  | 74 | 1.128 | 0.2862 | Yes |
| 29 | MCM4 |  |  | 81 | 1.110 | 0.2939 | Yes |
| 30 | POLA2 |  |  | 95 | 1.068 | 0.2994 | Yes |
| 31 | TMPO |  |  | 99 | 1.056 | 0.3074 | Yes |
| 32 | MCM3 |  |  | 105 | 1.035 | 0.3147 | Yes |
| 33 | MKI67 |  |  | 107 | 1.031 | 0.3230 | Yes |
| 34 | RFC2 |  |  | 112 | 1.022 | 0.3305 | Yes |
| 35 | CDCA8 |  |  | 113 | 1.018 | 0.3389 | Yes |
| 36 | TRIP13 |  |  | 114 | 1.014 | 0.3473 | Yes |
| 37 | DUT |  |  | 118 | 1.009 | 0.3549 | Yes |
| 38 | ASF1B |  |  | 120 | 1.007 | 0.3630 | Yes |
| 39 | DEPDC1 |  |  | 126 | 0.999 | 0.3701 | Yes |
| 40 | CKS1B |  |  | 128 | 0.999 | 0.3781 | Yes |
| 41 | TACC3 |  |  | 130 | 0.992 | 0.3861 | Yes |
| 42 | CDK4 |  |  | 137 | 0.986 | 0.3927 | Yes |
| 43 | RANBP1 |  |  | 149 | 0.958 | 0.3979 | Yes |
| 44 | DSCC1 |  |  | 155 | 0.946 | 0.4044 | Yes |
| 45 | CTPS1 |  |  | 162 | 0.934 | 0.4107 | Yes |
| 46 | DONSON |  |  | 163 | 0.933 | 0.4184 | Yes |
| 47 | RPA2 |  |  | 169 | 0.929 | 0.4248 | Yes |
| 48 | MCM6 |  |  | 176 | 0.906 | 0.4308 | Yes |
| 49 | CENPM |  |  | 178 | 0.901 | 0.4380 | Yes |
| 50 | DCLRE1B |  |  | 192 | 0.882 | 0.4420 | Yes |
| 51 | PLK4 |  |  | 205 | 0.870 | 0.4462 | Yes |
| 52 | CSE1L |  |  | 210 | 0.866 | 0.4524 | Yes |
| 53 | PRIM2 |  |  | 212 | 0.865 | 0.4593 | Yes |
| 54 | LYAR |  |  | 216 | 0.860 | 0.4657 | Yes |
| 55 | CDCA3 |  |  | 219 | 0.858 | 0.4723 | Yes |
| 56 | BUB1B |  |  | 223 | 0.851 | 0.4786 | Yes |
| 57 | EZH2 |  |  | 233 | 0.839 | 0.4832 | Yes |
| 58 | HMGA1 |  |  | 238 | 0.834 | 0.4891 | Yes |
| 59 | RACGAP1 |  |  | 241 | 0.831 | 0.4955 | Yes |
| 60 | NOP56 |  |  | 250 | 0.824 | 0.5003 | Yes |
| 61 | LMNB1 |  |  | 251 | 0.824 | 0.5072 | Yes |
| 62 | CHEK1 |  |  | 252 | 0.822 | 0.5140 | Yes |
| 63 | KIF18B |  |  | 254 | 0.815 | 0.5205 | Yes |
| 64 | DNMT1 |  |  | 265 | 0.792 | 0.5245 | Yes |
| 65 | HMGB2 |  |  | 276 | 0.780 | 0.5284 | Yes |
| 66 | SMC4 |  |  | 279 | 0.777 | 0.5344 | Yes |
| 67 | EIF2S1 |  |  | 282 | 0.776 | 0.5403 | Yes |
| 68 | CENPE |  |  | 284 | 0.775 | 0.5464 | Yes |
| 69 | BARD1 |  |  | 295 | 0.760 | 0.5502 | Yes |
| 70 | DEK |  |  | 302 | 0.754 | 0.5549 | Yes |
| 71 | POLE |  |  | 305 | 0.750 | 0.5606 | Yes |
| 72 | MMS22L |  |  | 306 | 0.747 | 0.5668 | Yes |
| 73 | KIF4A |  |  | 309 | 0.746 | 0.5725 | Yes |
| 74 | CKS2 |  |  | 312 | 0.745 | 0.5782 | Yes |
| 75 | SLBP |  |  | 318 | 0.740 | 0.5830 | Yes |
| 76 | TIPIN |  |  | 326 | 0.732 | 0.5873 | Yes |
| 77 | MTHFD2 |  |  | 337 | 0.721 | 0.5908 | Yes |
| 78 | SPC24 |  |  | 340 | 0.719 | 0.5962 | Yes |
| 79 | LIG1 |  |  | 341 | 0.718 | 0.6022 | Yes |
| 80 | RPA1 |  |  | 343 | 0.715 | 0.6079 | Yes |
| 81 | EED |  |  | 345 | 0.712 | 0.6135 | Yes |
| 82 | AURKA |  |  | 360 | 0.705 | 0.6158 | Yes |
| 83 | E2F8 |  |  | 379 | 0.691 | 0.6170 | Yes |
| 84 | PA2G4 |  |  | 412 | 0.672 | 0.6144 | Yes |
| 85 | GINS4 |  |  | 417 | 0.669 | 0.6190 | Yes |
| 86 | KPNA2 |  |  | 420 | 0.669 | 0.6240 | Yes |
| 87 | RAN |  |  | 424 | 0.667 | 0.6288 | Yes |
| 88 | SMC3 |  |  | 425 | 0.667 | 0.6343 | Yes |
| 89 | NCAPD2 |  |  | 457 | 0.647 | 0.6318 | Yes |
| 90 | MRE11 |  |  | 463 | 0.645 | 0.6359 | Yes |
| 91 | SNRPB |  |  | 467 | 0.642 | 0.6404 | Yes |
| 92 | HMMR |  |  | 475 | 0.639 | 0.6439 | Yes |
| 93 | DCTPP1 |  |  | 477 | 0.638 | 0.6490 | Yes |
| 94 | SSRP1 |  |  | 478 | 0.638 | 0.6543 | Yes |
| 95 | SHMT1 |  |  | 486 | 0.635 | 0.6578 | Yes |
| 96 | POP7 |  |  | 502 | 0.628 | 0.6592 | Yes |
| 97 | BIRC5 |  |  | 505 | 0.628 | 0.6639 | Yes |
| 98 | RPA3 |  |  | 532 | 0.611 | 0.6623 | Yes |
| 99 | DDX39A |  |  | 542 | 0.605 | 0.6651 | Yes |
| 100 | NOLC1 |  |  | 546 | 0.603 | 0.6693 | Yes |
| 101 | CDC25A |  |  | 572 | 0.589 | 0.6679 | Yes |
| 102 | IPO7 |  |  | 586 | 0.584 | 0.6694 | Yes |
| 103 | TIMELESS |  |  | 591 | 0.583 | 0.6732 | Yes |
| 104 | EXOSC8 |  |  | 604 | 0.576 | 0.6749 | Yes |
| 105 | TUBG1 |  |  | 617 | 0.570 | 0.6766 | Yes |
| 106 | SMC1A |  |  | 618 | 0.570 | 0.6813 | Yes |
| 107 | SPAG5 |  |  | 642 | 0.555 | 0.6801 | Yes |
| 108 | DCK |  |  | 661 | 0.548 | 0.6801 | Yes |
| 109 | PAICS |  |  | 668 | 0.546 | 0.6831 | Yes |
| 110 | NUP107 |  |  | 675 | 0.539 | 0.6860 | Yes |
| 111 | TUBB |  |  | 678 | 0.538 | 0.6900 | Yes |
| 112 | PRKDC |  |  | 681 | 0.537 | 0.6939 | Yes |
| 113 | WEE1 |  |  | 685 | 0.536 | 0.6976 | Yes |
| 114 | CIT |  |  | 690 | 0.535 | 0.7010 | Yes |
| 115 | NUP205 |  |  | 705 | 0.531 | 0.7019 | Yes |
| 116 | DLGAP5 |  |  | 712 | 0.529 | 0.7047 | Yes |
| 117 | POLD1 |  |  | 729 | 0.523 | 0.7050 | Yes |
| 118 | MCM2 |  |  | 734 | 0.521 | 0.7083 | Yes |
| 119 | NASP |  |  | 741 | 0.518 | 0.7111 | Yes |
| 120 | UNG |  |  | 750 | 0.516 | 0.7133 | Yes |
| 121 | MYC |  |  | 790 | 0.502 | 0.7076 | No |
| 122 | CDC20 |  |  | 844 | 0.486 | 0.6982 | No |
| 123 | UBR7 |  |  | 857 | 0.483 | 0.6992 | No |
| 124 | NUP153 |  |  | 865 | 0.480 | 0.7014 | No |
| 125 | PNN |  |  | 882 | 0.476 | 0.7012 | No |
| 126 | SUV39H1 |  |  | 925 | 0.464 | 0.6944 | No |
| 127 | RAD1 |  |  | 926 | 0.464 | 0.6983 | No |
| 128 | SRSF2 |  |  | 941 | 0.461 | 0.6986 | No |
| 129 | ZW10 |  |  | 958 | 0.457 | 0.6983 | No |
| 130 | RAD51C |  |  | 967 | 0.453 | 0.7000 | No |
| 131 | KIF22 |  |  | 975 | 0.450 | 0.7020 | No |
| 132 | PLK1 |  |  | 1009 | 0.442 | 0.6973 | No |
| 133 | CCNE1 |  |  | 1045 | 0.429 | 0.6919 | No |
| 134 | CHEK2 |  |  | 1073 | 0.424 | 0.6886 | No |
| 135 | NUDT21 |  |  | 1076 | 0.422 | 0.6916 | No |
| 136 | CDKN1A |  |  | 1088 | 0.419 | 0.6923 | No |
| 137 | CBX5 |  |  | 1132 | 0.412 | 0.6848 | No |
| 138 | HNRNPD |  |  | 1177 | 0.401 | 0.6770 | No |
| 139 | PHF5A |  |  | 1221 | 0.393 | 0.6693 | No |
| 140 | SMC6 |  |  | 1222 | 0.392 | 0.6726 | No |
| 141 | POLD2 |  |  | 1237 | 0.389 | 0.6722 | No |
| 142 | STMN1 |  |  | 1249 | 0.387 | 0.6726 | No |
| 143 | ESPL1 |  |  | 1255 | 0.386 | 0.6746 | No |
| 144 | POLE4 |  |  | 1281 | 0.381 | 0.6714 | No |
| 145 | CNOT9 |  |  | 1287 | 0.380 | 0.6733 | No |
| 146 | ANP32E |  |  | 1334 | 0.371 | 0.6647 | No |
| 147 | RNASEH2A |  |  | 1341 | 0.369 | 0.6662 | No |
| 148 | NME1 |  |  | 1343 | 0.368 | 0.6690 | No |
| 149 | AK2 |  |  | 1378 | 0.360 | 0.6634 | No |
| 150 | TBRG4 |  |  | 1415 | 0.353 | 0.6572 | No |
| 151 | PRDX4 |  |  | 1470 | 0.344 | 0.6463 | No |
| 152 | RFC1 |  |  | 1579 | 0.328 | 0.6216 | No |
| 153 | PRPS1 |  |  | 1620 | 0.320 | 0.6142 | No |
| 154 | GINS3 |  |  | 1694 | 0.312 | 0.5982 | No |
| 155 | MXD3 |  |  | 2156 | 0.256 | 0.4834 | No |
| 156 | XRCC6 |  |  | 2180 | 0.254 | 0.4797 | No |
| 157 | CDKN1B |  |  | 3812 | -0.800 | 0.0726 | No |
Table: GSEA details [plain text format]

  

Fig 2: HALLMARK\_E2F\_TARGETS: Random ES distribution      
 Gene set null distribution of ES for **HALLMARK\_E2F\_TARGETS**
